# The kinetics of islet amyloid polypeptide phase-separated system and hydrogel formation are critically influenced by macromolecular crowding

**DOI:** 10.1042/BCJ20210384

**Published:** 2021-08-13

**Authors:** Lior Pytowski, David J. Vaux, Létitia Jean

**Affiliations:** Sir William Dunn School of Pathology, University of Oxford, Oxford OX1 3RE, U.K.

**Keywords:** amyloid, gelation, IAPP, macromolecular crowding, phase separation

## Abstract

Many protein misfolding diseases (e.g. type II diabetes and Alzheimer's disease) are characterised by amyloid deposition. Human islet amyloid polypeptide (hIAPP, involved in type II diabetes) spontaneously undergoes liquid–liquid phase separation (LLPS) and a kinetically complex hydrogelation, both catalysed by hydrophobic–hydrophilic interfaces (e.g. air–water interface and/or phospholipids–water interfaces). Gelation of hIAPP phase-separated liquid droplets initiates amyloid aggregation and the formation of clusters of interconnected aggregates, which grow and fuse to eventually percolate the whole system. Droplet maturation into irreversible hydrogels via amyloid aggregation is thought to be behind the pathology of several diseases. Biological fluids contain a high volume fraction of macromolecules, leading to macromolecular crowding. Despite crowding agent addition in *in vitro* studies playing a significant role in changing protein phase diagrams, the mechanism underlying enhanced LLPS, and the effect(s) on stages beyond LLPS remain poorly or not characterised.We investigated the effect of macromolecular crowding and increased viscosity on the kinetics of hIAPP hydrogelation using rheology and the evolution of the system beyond LLPS by microscopy. We demonstrate that increased viscosity exacerbated the kinetic variability of hydrogelation and of the phase separated-aggregated system, whereas macromolecular crowding abolished heterogeneity. Increased viscosity also strengthened the gel meshwork and accelerated aggregate cluster fusion. In contrast, crowding either delayed cluster fusion onset (dextran) or promoted it (Ficoll). Our study highlights that an *in vivo* crowded environment would critically influence amyloid stages beyond LLPS and pathogenesis.

## Introduction

The pathology of many degenerative diseases, such as Alzheimer's disease and type 2 diabetes mellitus (D2M), is strongly associated with the deposition of insoluble proteinaceous aggregates termed amyloids [1]. The burden of these diseases on human health is far-reaching, which has triggered a great deal of research into the understanding of the complexity of amyloid formation. In D2M, the misfolding of islet amyloid polypeptide (IAPP), a 37 amino acid peptide, has been implicated in the degeneration of the pancreatic islets of Langerhans [2]. IAPP is co-expressed and co-secreted with insulin from the β cells and, in D2M, the loss of insulin control coincides with the deposition of IAPP aggregates and the loss of β cell mass.

Despite the absence of sequence homology between amyloid precursors, their aggregation follows a similar nucleation-dependent polymerisation process [3]. First, monomers undergo an energetically unfavourable nucleation step (lag phase, β-sheet formation), before the nuclei elongate by monomer addition until the system reaches equilibrium between monomers and fibrils (plateau phase). Due to the amphiphilicity of many amyloid polypeptides, amyloidogenesis can be catalysed by hydrophilic–hydrophobic interfaces (HHIs) both *in vitro* (air–water interface, AWI, non-polar gas, and polar aqueous solution) and *in vivo* (phospholipid membranes, non-polar fatty acid tails and aqueous environment, or charged phospholipid headgroups) [4–11]. Adsorption to HHIs allows amyloid polypeptides to spatially concentrate, to align their side chains, to promote β-sheet formation and ultimately to assemble into amyloid species [10–15]. In turn, amyloid aggregation at membranes leads to membrane damage and is postulated to be an important cause of amyloid toxicity [16–18]. IAPP interacts with β cell membranes, which leads to a concomitant increase in membrane permeability and calcium dysregulation [18,19].

Many proteins can undergo liquid–liquid phase separation (LLPS) and/or form hydrogels. These processes have also been reported for a variety of amyloid polypeptides [20–29]. In recent years, LLPS has received a lot of attention, as not only it is responsible for the formation of membrane-less organelles, but also because it may be associated with pathology due to droplet maturation into irreversible hydrogels via amyloid-like aggregation [22,29–31]. We previously demonstrated by rheology the formation of a 3D hydrogel by full-length non-mutated human IAPP, consisting of a 3D supramolecular network of condensed fibrils, which was initially catalysed by the AWI or phospholipids [32]. Two distinct kinetic regimes led to IAPP hydrogelation, suggesting the existence of multiple gelation pathways. Very recently, we showed that IAPP, without any added triggers (e.g. macromolecular crowding), can spontaneously undergo an AWI-catalysed LLPS, with the initial liquid droplets maturing into a hydrogel-state followed by amyloid aggregation at the droplet surface. The aggregates further grew to create a macroscopic network of interconnected aggregate clusters, which evolved into a gelled meshwork and propagated into the bulk. LLPS explains the kinetic variation of IAPP hydrogelation, during which the system can evolve independently from multiple pathways, each generated from varying number of droplets. The consequences of LLPS and hydrogelation during amyloidogenesis could be really critical in term of membrane integrity and overall cellular functions.

*In vivo*, biological fluids are occupied by a high concentration of macromolecules, which non-specifically and sterically exclude other molecules, in a process called macromolecular crowding [33,34]. Protein stability, folding, and protein–protein interactions are all affected, either kinetically or thermodynamically, by this volume exclusion [35,36]. Volume exclusion is not the only effect of macromolecular crowding as it can also cause an increase in viscosity, changes in protein solvation, water content, and solvent polarity [34,36,37]. Aggregation (including amyloidogenesis) can be promoted by volume exclusion, resulting in protein destabilisation and a shift in macromolecular equilibria [36,37]. However, macromolecular crowding-induced increase in viscosity can also reduce amyloidogenic monomer diffusion to sites of assembly (AWI or fibril ends, for example) and therefore alter the kinetics of amyloidogenesis [34,37,38]. *In vitro*, amyloidogenesis is mostly studied in dilute conditions and when studied under crowding conditions, it is mainly fibrillisation reactions that have been investigated [37–42]. These studies showed that amyloidogenesis may either be enhanced due to volume exclusion or inhibited due to a viscosity increase resulting in a decrease of the rate of fibrillisation.

Despite crowding agents being used ubiquitously in almost all *in vitro* studies to trigger LLPS, their effect on the kinetics of phase-separating systems or the effect of different types of crowder have rarely been investigated or even discussed. Macromolecular crowding affects LLPS; for many proteins, the critical concentration required for LLPS is reduced by crowder addition, and LLPS may not occur at all in the absence of a crowding agent [28,43,44]. However, the mechanism(s) behind crowding-induced LLPS are mostly unknown. Furthermore, despite the liquid to gel transition of phase-separated droplets triggering pathological aggregation, very few studies have investigated macromolecular crowding in the context of stages beyond LLPS. This understanding is relevant to the study of LLPS and subsequent stages *in vivo*, where macromolecular crowding is prominent, in particular when LLPS-driven aggregation is thought to be at the heart of some pathologies.

In this study, we investigated the effect of macromolecular crowding, by dextran 70 kDa and Ficoll 400 kDa, in driving and modulating the stages beyond LLPS of full-length hIAPP. The rational for investigating stages beyond LLPS was two-fold. First, whilst crowding is a well-known promoter of LLPS so its effect is already known despite of the mechanism behind it remaining poorly characterised. Second, the stages beyond LLPS (aggregation, aggregate cluster formation, and hydrogelation of the whole system) are thought to be important for the development of pathologies, and are rarely studied in the context of crowding. We characterised the kinetics of IAPP hydrogelation using rheology and the evolution of the system beyond LLPS by microscopy. We show that the previously described kinetics variability of IAPP hydrogelation was exacerbated by viscosity increase (by glycerol), which also changed the properties of the gelled network, but was abolished by macromolecular crowding. We also demonstrate that increased viscosity exacerbated the heterogeneity of the phase separated-aggregated system and aggregate cluster fusion, altered the gel meshwork and accelerated cluster fusion. In contrast, both dextran and Ficoll abolished the heterogeneity of the phase separated-aggregated system, giving rise to a delayed but more kinetically homogenous aggregate cluster fusion. However, the effects of these two crowding agents were not identical since dextran delayed the onset of fusion and fusion itself, whereas Ficoll promoted fusion onset and delayed fusion itself. These results offer a more detailed understanding of the effect of macromolecular crowding on stages following LLPS, aggregation, and hydrogelation, which are critical for pathology. Indeed, the transition of phase-separated liquid droplets to a gel-like state promotes pathological aggregation (e.g. fused in sarcoma, FUS, in amyotrophic lateral schlerosis) [30].

## Materials and methods

### Peptides and reagents

Lyophilised synthetic human IAPP (Bachem AG supplied by Cambridge Bioscience, Cambridge, Cambridgeshire, U.K.) was purchased already purified by reverse-phase high-performance liquid chromatography. IAPP was resuspended in DMSO at 512.4 μM, sonicated and centrifuged for 1 h at 15000 g at + 4°C prior to use (to remove any pre-aggregated species). DMSO was used to maintain IAPP in a monomeric pool lacking any β-sheet secondary structures [45]. Stock solutions of 300 g/l of dextran (64–76 kDa) (Sigma–Aldrich Merck, Gillingham, Dorset, U.K.) and Ficoll (400 kDa) (Sigma–Aldrich), and 30% glycerol (VWR, Luttrworth, Leicestershire, U.K.) were prepared in distilled water.

### Rheology

Measurements were performed, at 25°C, on a Bohlin Gemini 200 HR Nano rheometer (Malvern Instruments, Malvern, Worcestershire, U.K.). 4 μM IAPP (from the DMSO stock solution) was pipetted onto the lower plate of the rheometer and, when required, 6% glycerol, or 6% Ficoll400, or 6% dextran70 were also pipetted onto the lower plate but independently to IAPP (to preclude premature mixing). Water was then added to get a 1.4 ml reaction volume before the upper plate (measuring cone geometry, *D *= 40 mm, 4° incline) was lowered as slowly as possible onto the sample to ensure a completely filled gap. An environmental cuff with moistened tissue inside was placed around the geometry to prevent sample dehydration. Controls containing DMSO, or 6% glycerol, or 6% Ficoll400, or 6% dextran70 with DMSO were also performed. Oscillation time sweeps were recorded with a controlled displacement of 5 × 10^−3^ rads and a frequency of 0.5 Hz. Frequency sweeps were also performed. For IAPP alone, six independent experiments were performed [32]; for IAPP in presence of 6% glycerol, four independent experiments were performed; and for IAPP in presence of dextran or Ficoll, three independent experiments were performed. Statistical analysis was performed with the two-sample *t*-test.

### Aggregate clustering and connectedness

3.6 μM IAPP–0.4 μM bIAPP–0.08 μM avidin D with 6% glycerol, or 6% dextran70 or 6% Ficoll400 in PBS in a 345 μl reaction volume, was pipetted in a well of a 96-well plate (black wall, clear bottom). The experimental setup has previously been described [29]. Briefly, the well was sealed and placed upside down onto the stage plate holder of Zeiss LSM 880 Confocal Microscope, within the controlled chamber setup at 25°C. The well was imaged with a 10× objective, and images acquired using ZEN operating software. *z* stacks encompassing the AWI and an area spanning 1417 × 1417 μm^2^, from the edge of the well, were collected with a 5 μm interval between *z* slices and with no time gap between stacks for at least 15 h. 3D reconstruction was performed in Imaris v8.4.1, and visualised with the AWI at the front facing the experimentalist and the bulk solution behind it. Maximum intensity *z*-projections were made in FIJI [46,47]. Particle tracking, and analysis of particle trajectories, mean aggregate speed and aggregate connectivity have been previously described [29].

## Results

*In vivo*, macromolecular crowding occurs from the presence of various molecules with different properties, shapes, lengths, and molecular mass. Cellular ‘crowded’ conditions can be mimicked *in vitro* by the addition of non-specific model crowding agents [34,48]. The most common crowding agents used to promote LLPS are Ficoll, dextran, and PEG. However, the size, molecular mass, and the nature of the crowding agents should be considered as they can impact LLPS differently, as was previously shown for lysozyme or pentamers of SH3 domain-proline rich motifs [49,50]. Thus, we investigated the impact of polymer crowders with different properties and chain length, dextran70 (70 kDa) and Ficoll400 (400 kDa). Both polymers are non-ionised, hydrophilic, more closely related to the types of macromolecules found physiologically in a cellular environment, and importantly do not interact with proteins and IAPP [42,51]. In contrast with dextran, Ficoll creates macromolecular crowding with relatively low viscosity [38]. Therefore, comparison between these crowding agents permits assessment of the contribution of viscosity to macromolecular crowding responses. However, Ficoll is also surface active, whereas dextran is not and dextran promotes a higher volume occupancy.

### Increased viscosity introduces further kinetic variability in IAPP hydrogelation whereas macromolecular crowding abolishes it

**Figure 1.**
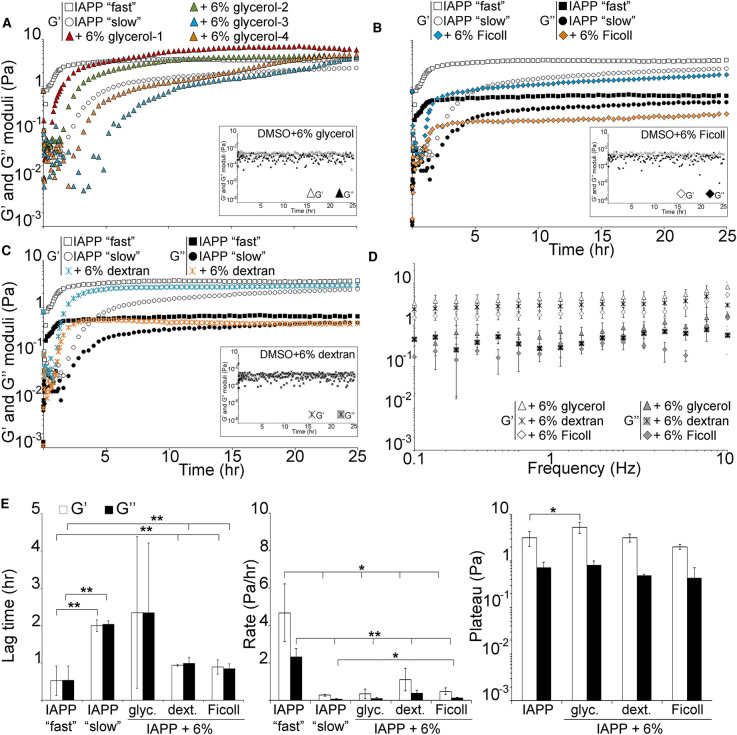
3D gelation of IAPP is affected by viscosity and macromolecular crowding. Rheological properties of a 4 μM IAPP solution in the absence or presence of 6% glycerol (**A**), Ficoll (**B**), or dextran (**C**) were assessed at 25°C with a controlled displacement of 5 × 10^−3^ rads and a frequency of 0.5 Hz. Dynamic moduli G′ and G″ as a function of time, with the *insets* showing appropriate DMSO controls. Only G′ is shown in (**A**) for clarity. (**D**) G′ and G″’ as a function of angular frequency *ω*. (**E**) The lag time for moduli increase, rate of increase and plateau reached are depicted. The mean of at least three independent assays is shown, except in (**A**) where four independent assays are shown for IAPP in presence of 6% glycerol. Error bars represent ± SEM. * *P *< 0.05 and ** *P *< 0.03 when compared with IAPP alone.

We previously demonstrated that the kinetics of IAPP hydrogelation followed complex dynamics with two distinct kinetic regimes (‘slow’ and ‘fast’; Figure 1), each gelation pathway generated from varying number of phase-separated droplets and evolving independently [29,32]. The hydrogelation onset (lag time for moduli increase) was ∼2 h for the ‘slow’ regime and ∼0.5 h for the ‘fast’ regime (with an average of 1.26 ± 0.85 h), and full hydrogelation (beginning of G′ plateau) had occurred at 22 and ∼3 h, respectively, to reach a G′ plateau of ∼3.6 Pa for both regimes [32]. The moduli increased at 0.28 (G′) and 0.08 (G’) Pa/h for the ‘slow’ regime, and 4.7 (G′) and 2.3 (G’) Pa/h for the ‘fast’ one.

Here, we investigated further IAPP gelation by using macromolecular crowding conditions that could affect the process and mirror a physiological environment, macromolecular crowding. Since the crowders dextran and Ficoll are viscous polymers, we also investigated the effect of viscosity alone by using glycerol, which is a small polyol osmolyte having negligible crowding effects. We used 6% of crowders or glycerol as we previously showed that this amount of crowder had a maximum inhibiting effect on fibrillisation without fully abolishing it, in particular for Ficoll [38].

Overall, glycerol did not statistically delay the onset of IAPP gelation (an average of 2.35 ± 2.03 h). However, two independent replicates out of four were slower than both the ‘slow’ and ‘fast’ IAPP regimes (Figure 1A,E, Supplementary Figures S1 and S2). The average rate of gelation was decreased by glycerol (0.36 ± 0.26 Pa/h for G′, *P *< 0.039 when compared with the ‘fast’ IAPP regime), and was similar to that of the ‘slow’ regime. However, in glycerol, the G′ plateau (5.31 ± 1.45 Pa) was ∼1.5 fold higher than that of IAPP alone (*P* < 0.049). Glycerol by itself did not affect the rheological measurements (Figure 1A, insets). Indeed, G′ dominated G″ at all frequencies tested with the Tan value lower than 1 and the moduli showing frequency independence (Figure 1D). Moreover, the graph of system variation (SD over mean) for G′ revealed that the kinetic variation in presence of glycerol was mostly higher than that of IAPP alone, for a longer time period, maximal before gelation started and remained (<5 h) large until gelation had reached equilibrium (G′ plateau, ∼23 h) (Supplementary Figure S3A). Therefore, the system with glycerol was more heterogeneous than that without.

In the presence of dextran or Ficoll (three independent experiments for each), the variation in kinetics for IAPP gelation (both time and rate) was abolished with the system settling down for an intermediate regime between ‘slow’ and ‘fast’ (Figure 1B, C and E, Supplementary Figure S2). The onset of hydrogelation was <1 h with both dextran (0.94 ± 0.03 h) and Ficoll (0.89 ± 0.19 h), which was ∼2.1–2.2 fold faster than that of IAPP ‘slow’ regime (*P *< 0.007) but 1.8–1.9 fold slower than IAPP ‘fast’ regime. The rate of G′ and G″ increase were significantly slower than that of IAPP ‘fast’ regime (respectively, 1.12 ± 0.61 and 0.39 ± 0.16 Pa/h with dextran, and 0.50 ± 0.16 and 0.15 ± 0.03 Pa/h with Ficoll; *P* < 0.043 for G′ and *P *< 0.016 for G″). The plateau for G′ (3.15 ± 0.57 Pa with dextran, and 2.00 ± 0.25 Pa with Ficoll) was not significantly different to that of IAPP alone. The crowders by themselves did not affect the rheological measurements (Figure 1B,C, insets). Indeed, G′ dominated G″ at all frequencies tested with the Tan value lower than 1 and the moduli showing frequency independence (Figure 1D). With dextran and Ficoll, the overall system variation (graph of SD over mean for G′) was much lower and for a shorter time period than that of IAPP alone and the rheological curves for all replicates over time were almost identical, demonstrating that gelation occurred concomitantly in all replicates and the system kinetics were homogeneous (Supplementary Figure S3).

Altogether, these results are characteristic of the formation of a 3D hydrogel by the entire IAPP solution (bulk and AWI) independently of the macromolecules added. However, it is clear that the gelation kinetic regimes adopted were highly dependent upon viscosity or macromolecular crowding.

### At the AWI, increased viscosity exacerbates the heterogeneity of the phase separated and aggregated system

We previously showed that IAPP spontaneously underwent AWI-catalysed LLPS, with the droplets undergoing hydrogelation, which triggered aggregation from the droplet surface [29]. At the AWI, the phase-separated aggregates grew into clusters of interconnected aggregates, which fused together to create a macroscopic network percolating the whole system [29]. Macromolecular crowding is one of the main tool used to trigger LLPS *in vitro*. However, the effect of crowding on the evolution of the system beyond the droplet stage has never been studied. As a direct reporter of IAPP assembly, we used biotinyl IAPP (bIAPP,1 : 9 to IAPP) and avidin D fluorescein, which has previously been shown to be a good reporter of IAPP LLPS [29].

**Figure 2.**
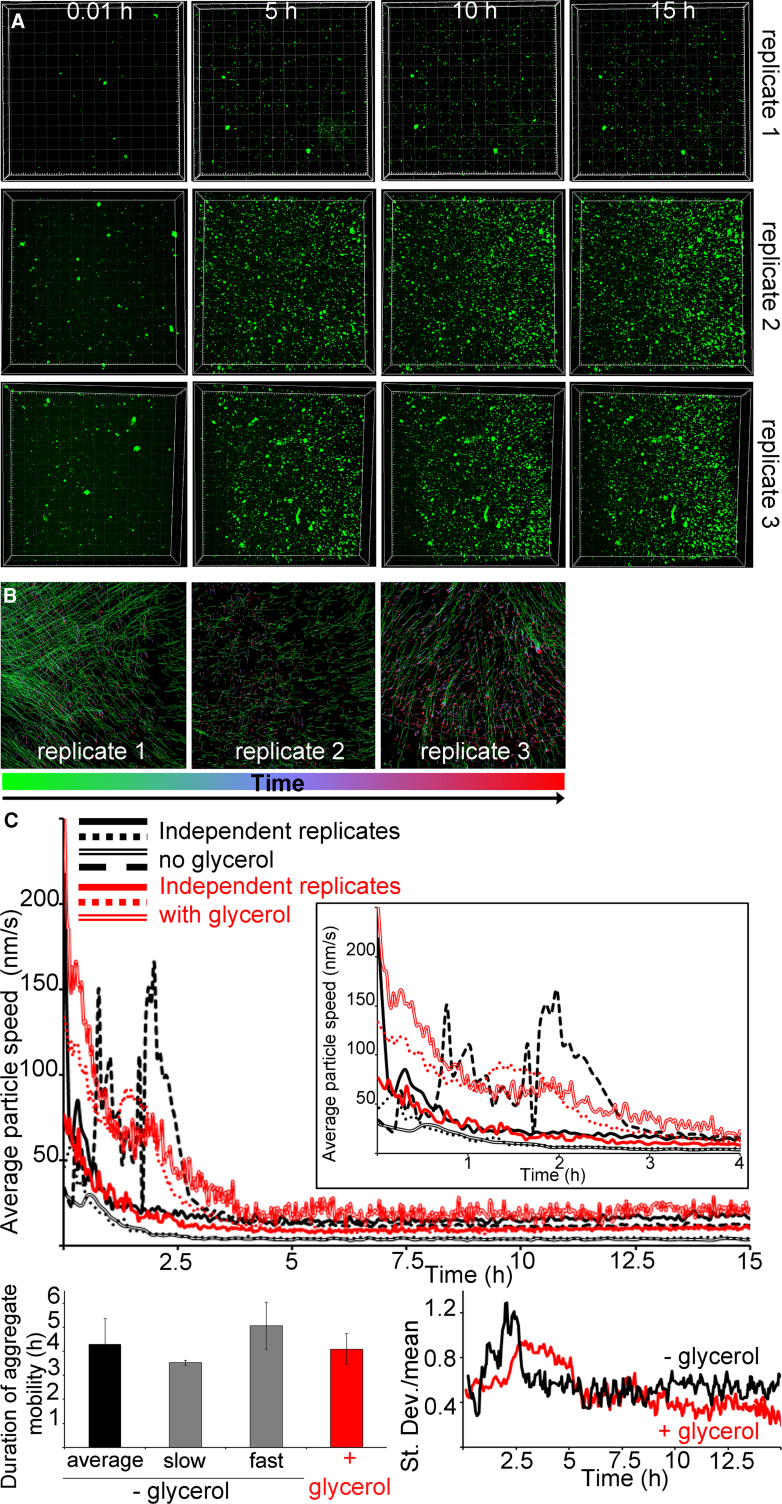
Increased viscosity exacerbates the heterogeneity of the IAPP phase separated and aggregated system at the AWI. (**A**) In presence of glycerol, IAPP aggregate accumulation at the AWI is heterogeneous. 3.6 μM IAPP–0.4 μM bIAPP–0.08 μM avidin D fluorescein was incubated with 6% glycerol in a well of a 96-well plate. The well was imaged, *z* stacks encompassing the AWI and an area spanning 1417 × 1417 μm^2^ were collected for at least 15 h. 3D projections were performed in Imaris and visualised with the AWI at the front facing the experimentalist and the bulk solution behind it. Presented are snapshots of 3D projections at relevant time points for three replicates. See also Supplementary Movie S1. (**B**) Tracking of the aggregates and their trajectories were performed for reactions described in (**A**). Shown are aggregate trajectories for three replicates. (**C**) Glycerol exacerbates the system heterogeneity, in particular around gelation onset. For the reactions described in (**A**), the speed of aggregates was calculated, along with that of the mean flow. Shown are the aggregate speed with that of mean flow subtracted overtime (top panel). The insets show a zoom up of early time points. The duration of aggregate mobility has been calculated from the top panel and is depicted at the bottom left. The coefficient of variation (standard deviation over mean) as a function of time is also shown (bottom right), i.e. to depict the variation between the reactions. Error bars represent ± SEM.

First, we investigated the effect of viscosity by using live microscopy with a setup previously validated [29]. In presence of glycerol, highly mobile hIAPP–bIAPP avidin fluorescein aggregates accumulated over time at the AWI, before becoming progressively immobile, as was previously reported for IAPP alone in water (Figure 2A, Supplementary Movie S1) [29]. The increased aggregate immobility was due to aggregate entrapment within a forming hydrogel in the whole system. In presence of glycerol at 15 h, the system was heterogeneous as the aggregate number and distribution between replicates was different.

To determine the timing of hydrogelation, we tracked the aggregates at the AWI, determined their trajectories, and quantified the average aggregate speed and duration of aggregate mobility. Using this analysis for bIAPP by itself in water, aggregates were previously found to undergo movement at early time points, the average speed of aggregates to be heterogeneous at times preceding gelation, and the averaged gelation time to be 4.29 ± 1.05 h [29]. It has to be noted that hydrogelation by bIAPP fell into two kinetic regimes, a ‘fast’ regime with two independent replicates taking 3.45 and 3.59 h to form a hydrogel (3.5 h on average), and a ‘slow’ regime with another two independent replicates taking 4.38 and 5.75 h (5.1 h on average). The existence of two regimes was also observed by rheology (Figure 1).

In the presence of glycerol, the aggregate movement also occurred mostly at early time points (green vs red traces), and the average aggregate speed over time varied between replicates, in particular up to 5 h, but to a lesser extent than for bIAPP alone (Figure 2B,C). Gelation of the whole system took on average 4.08 ± 0.64 h, which was not significantly different to the averaged timing for bIAPP alone. Hydrogelation was heterogeneous as the three replicates had different timings, 3.37, 4.30, and 4.58 h. By rheology, IAPP hydrogelation in presence of glycerol was also heterogeneous and occurred in the whole system (i.e. beginning of G′ plateau) ∼3.6 to 10.8 h (Figure 1). The timings seen here fall within this range. The variation graph (SD over mean) showed that the system was indeed heterogeneous, that the variation spanned a wider time range and occurred later than for bIAPP alone, and that the variation was maximal from just prior to just after the onset of gelation (2.5–5 h). Therefore, glycerol exacerbated the system heterogeneity, in particular around gelation onset, but did not interfere with gelation of the whole system.

### At the AWI, macromolecular crowding abolishes heterogeneity of the phase separated and aggregated system

**Figure 3.**
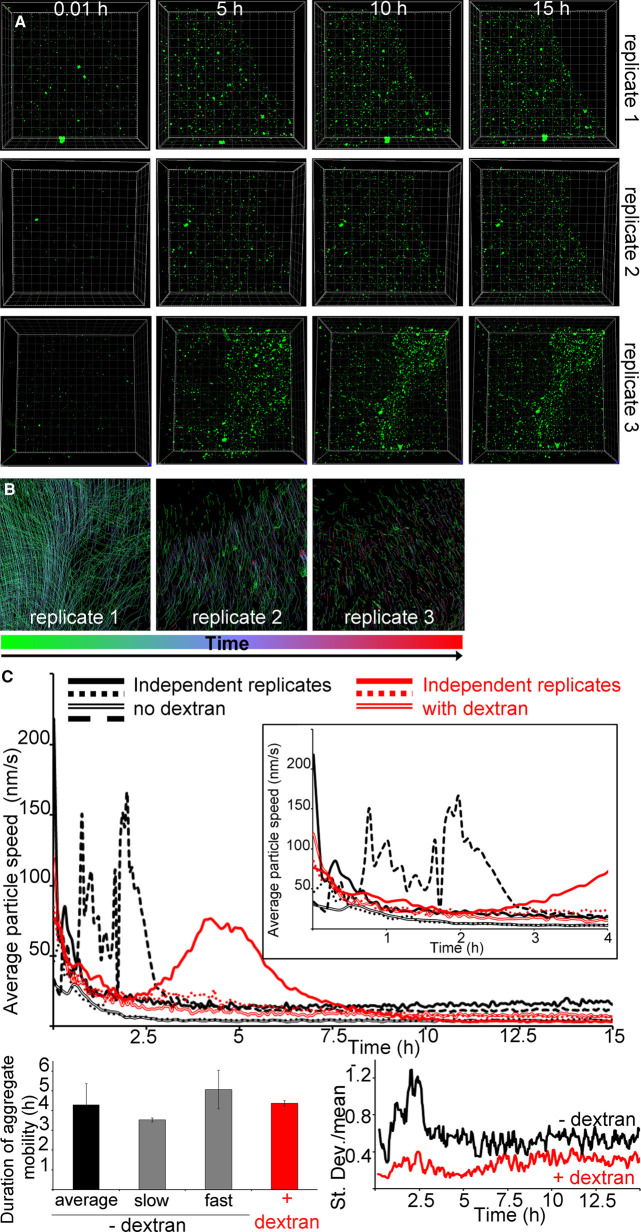
Macromolecular crowding with dextran abolishes the heterogeneity of IAPP phase separated and aggregated system at the AWI. (**A**) In presence of dextran, IAPP aggregate accumulation at the AWI is homogeneous. 3.6 μM IAPP–0.4 μM bIAPP–0.08 μM avidin D fluorescein was incubated with 6% dextran in a well of a 96-well plate. The well was imaged, *z* stacks encompassing the AWI and an area spanning 1417 × 1417 μm^2^ were collected for at least 15 h. 3D projections were performed in Imaris and visualised with the AWI at the front facing the experimentalist and the bulk solution behind it. Presented are snapshots of 3D projections at relevant time points for three replicates. See also Supplementary Movies S2 and S4. (**B**) Tracking of the aggregates and their trajectories were performed for reactions described in (**A**). Shown are aggregate trajectories for three replicates. (**C**) Dextran abolished the system heterogeneity, in particular around gelation onset. For the reactions described in (**A**), the speed of aggregates was calculated, along with that of the mean flow. Shown are the aggregate speed with that of mean flow subtracted overtime (top panel). The insets show a zoom up of early time points. The duration of aggregate mobility has been calculated from the top panel and is depicted at the bottom left. The coefficient of variation (standard deviation over mean) as a function of time is also shown (bottom right), i.e. to depict the variation between the reactions. Error bars represent ± SEM.

**Figure 4.**
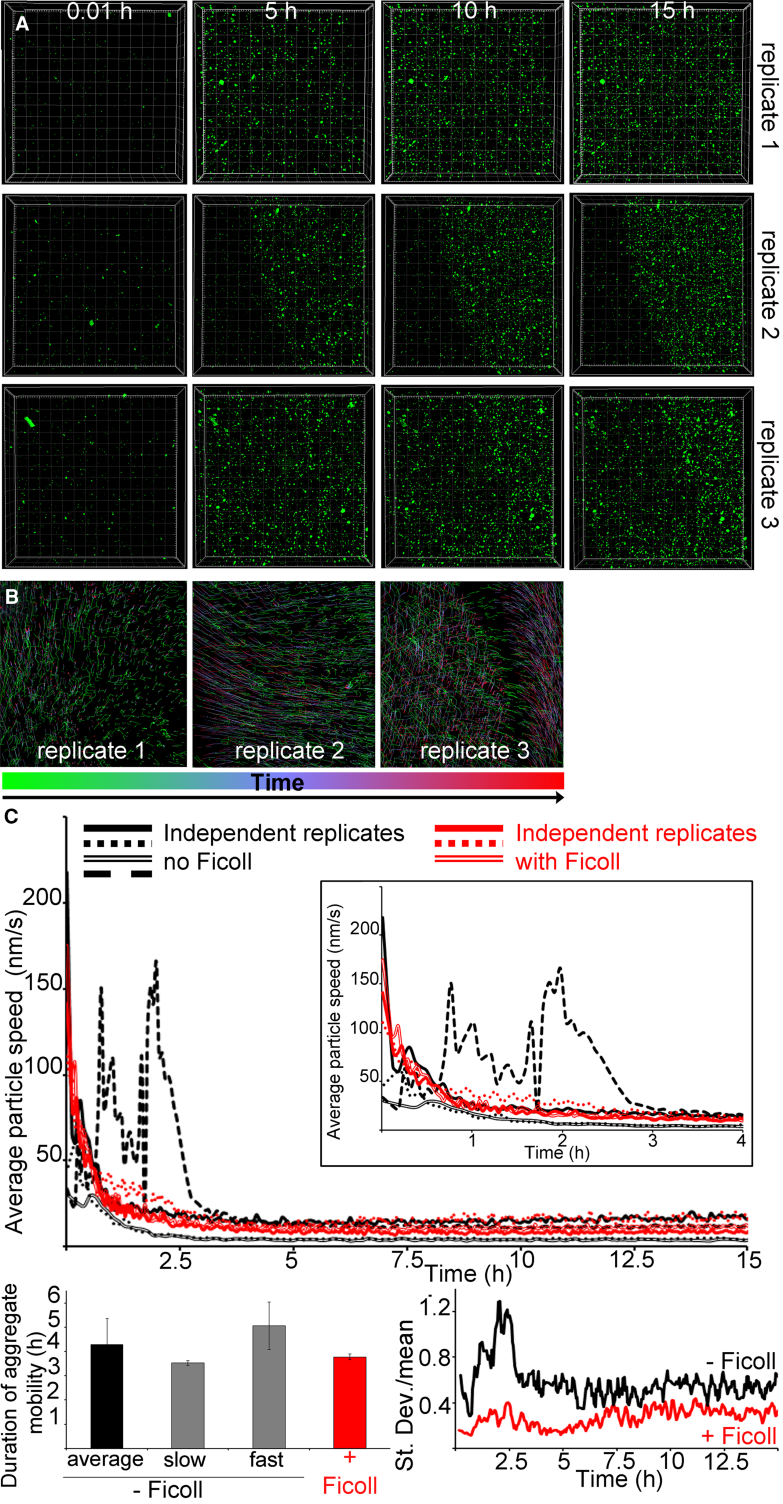
Macromolecular crowding with Ficoll abolishes the heterogeneity of IAPP phase separated and aggregated system at the AWI. (**A**) In presence of Ficoll, IAPP aggregate accumulation at the AWI is homogeneous. 3.6 μM IAPP–0.4 μM bIAPP–0.08 μM avidin D fluorescein was incubated with 6% Ficoll in a well of a 96-well plate. The well was imaged, *z* stacks encompassing the AWI and an area spanning 1417 × 1417 μm^2^ were collected for at least 15 h. 3D projections were performed in Imaris and visualised with the AWI at the front facing the experimentalist and the bulk solution behind it. Presented are snapshots of 3D projections at relevant time points for three replicates. See also Supplementary Movie S3. (**B**) Tracking of the aggregates and their trajectories were performed for reactions described in (**A**). Shown are aggregate trajectories for three replicates. (**C**) Ficoll abolishes the system heterogeneity, in particular before gelation started. For the reactions described in (**A**), the speed of aggregates was calculated, along with that of the mean flow. Shown are the aggregate speed with that of mean flow subtracted overtime (top panel). The insets show a zoom up of early time points. The duration of aggregate mobility has been calculated from the top panel and is depicted at the bottom left. The coefficient of variation (standard deviation over mean) as a function of time is also shown (bottom right), i.e. to depict the variation between the reactions. Error bars represent ± SEM.

Then we investigated the effect of macromolecular crowding on the whole system beyond droplet formation. In the presence of dextran or Ficoll, highly mobile hIAPP–bIAPP avidin fluorescein aggregates accumulated over time at the AWI, before becoming progressively immobile (Figures 3A and 4A, Supplementary Movies S2 and S3). For dextran, aggregate number and distribution were similar for replicates 1 and 2, whereas the aggregate distribution in replicate 3 appeared different with the aggregates being more compact. This compaction was due to gelation of the system outside of the field of view compressing the gelled system in the field of view (Supplementary Movie S4). Therefore, the compaction was ‘forced’ rather than reflecting a true difference in aggregate distribution. Thus, overall, crowding introduced by dextran also abolished the system heterogeneity. Macromolecular crowding introduced by Ficoll abolished the system heterogeneity, as aggregate number and distribution between replicates was similar. Aggregate tracking at the AWI revealed that aggregate movement occurred mostly early (at the green end of the time-dependent lookup table) but that co-ordinated all in one movement of the whole system could occur at later time points (blue-red, due to gelation outside of the field of view affecting the system in the field of view) (Figures 3B and 4B).

In the presence of dextran, the aggregates in all replicates displayed similar average speed over time and similar gelation times (Figure 3C). One of the replicate (replicate 3) displayed a ‘bump’ in the average speed curve after gelation onset, which was due to gelation of the system outside of the field of view as explained above (Supplementary Movie S4). This clearly occurred after gelation onset and therefore did not interfere with the following analysis. Gelation of the whole system took 4.37 ± 0.13 h, similarly to the average for bIAPP alone. As found rheologically, gelation with dextran settled for a unique regime intermediate between ‘slow’ and ‘fast’. The variation graph (SD over mean) showed that dextran abolished the system heterogeneity, in particular before gelation started.

In the presence of Ficoll, the aggregates in all replicates displayed similar average speed over time and similar gelation times (Figure 4C). Gelation of the whole system took 3.77 ± 0.12 h, faster by 1.14 fold than for the average of bIAPP alone, but not statistically different. As found rheologically, gelation with Ficoll settled for a unique regime intermediate between ‘slow’ and ‘fast’. The variation graph (SD over mean) showed that Ficoll, like dextran, abolished the system heterogeneity, in particular before gelation started.

### At the AWI, interconnected aggregates form clusters, which grow and fuse together, but cluster fusion is heterogeneous

We then investigated whether aggregates within a cluster were connected to each other and moving co-ordinately. Previously, we showed that aggregates moved together in local flow fields to form interconnected clusters, the clusters then grew over time and fused together to form bigger clusters [29]. We found that mostly bIAPP assays in D_2_O showed multiple, well defined, and easy to identify cluster fusions, however, aggregate clusters were also observed for bIAPP assays in H_2_O, although less abundant. Therefore, we further investigated these.

**Figure 5.**
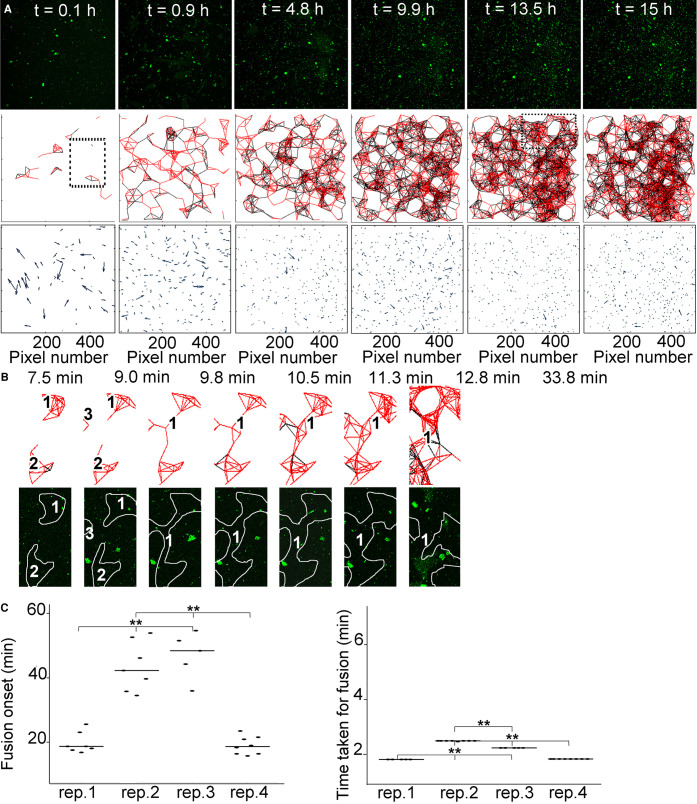
At the AWI, connected IAPP aggregates move co-ordinately in local flow fields, form individual clusters that grow and fuse together, to finally form a gelled meshwork. 3.6 μM IAPP–0.04 μM bIAPP–0.08 μM avidin D fluorescein in PBS was incubated in a well of a 96-well plate. The well was imaged, *z* stacks encompassing the AWI collected for at least 15 h, maximum intensity projections of the time course and aggregate tracking performed. From the aggregate tracking, movement vectors and connectivity between aggregates were determined. In (**A**) are shown maximum intensity projections of relevant time points of an 15 h time course (top panels), the connectivity between aggregates (a red line depicts aggregates moving in the same direction, i.e. connectedness; and a black line depicts aggregates moving in the opposite direction, i.e. non-connectedness) (middle panels), and the movement vectors (bottom panels). The dashed box is an area further examined in (**B**). (**B**) shows an example of aggregate connectivity over relevant time points (top panels), and the corresponding snapshots from a maximum intensity projection (bottom panels). The numbers within the projections indicate individual clusters of connected aggregates that evolve over time, and the white lines show the clusters of connected aggregates (the projection and connectedness were superimposed and lines were drawn around connected aggregates). (**C**) Quantitation of the timing taken for fusion event to occur (left panel) and time taken for fusion to be complete (right panel). Four independent replicates (rep.) have been analysed, with multiple fusion events per replicate. ** *P *< 0.0006.

In H_2_O, at early time points (e.g. 0.1 and 0.9 h), several individual connected aggregate clusters of bIAPP could be observed, as previously described (Figure 5A). As the reaction evolved, the original individual clusters grew before starting to fuse together (e.g. 4.8 h; larger clusters with increase connectedness). At later time points (e.g. 13.5 and 15 h), the clusters grew even further and interconnected with each other even more, to finally generate a macroscopic network containing connected but dispersed aggregate clusters. We then looked more closely at the dynamics of cluster growth and fusion. Although, cluster fusion was less well defined and thus more difficult to identify than that in D_2_O, we were nonetheless able to observe this behaviour. In the example of Figure 5B, two to three individual aggregate clusters could clearly be seen. These clusters grew (from 7.5 to 9.0 min) before fusing together relatively rapidly (∼0.8 min, from 9.0 to 9.8 min) to form a bigger cluster of interconnected aggregates, which carried on growing and evolving.

Finally, we did a systematic quantitation of the timing of fusion of aggregate clusters and of the time taken for fusion to be complete (Figure 5C). For this, we analysed four independent replicates and several individual aggregate clusters and fusion events per replicate. Similarly to hydrogelation (both by rheology and microscopy), the onset of the first cluster fusion event (on average 26.9 ± 16.9 min) and the time taken for fusion to be completed (on average 1.11 ± 0.35 min) were heterogeneous, with two kinetic regimes clearly identified and statistically different to one another (*P *< 0.0006). The ‘slow’ regime included replicates 1 and 4 (on average 41.5 min for fusion onset and 1.41 min for fusion completion), and the ‘fast’ regime included replicates 2 and 3 (on average 11.14 min for fusion onset and 0.85 min for fusion completion). Furthermore, for fusion onset, we observed variation between replicates but also some within replicates, suggesting an overall heterogeneity of the kinetics mostly between but also some within replicates. In contrast, the time taken for fusion to occur varied between replicates but showed no variation within replicates. This suggests that once fusion was underway, it occurred very quickly and homogeneously.

### At the AWI, the herogeneity of cluster fusion is exacerbated by glycerol, and glycerol interferes with the fusion process

**Figure 6.**
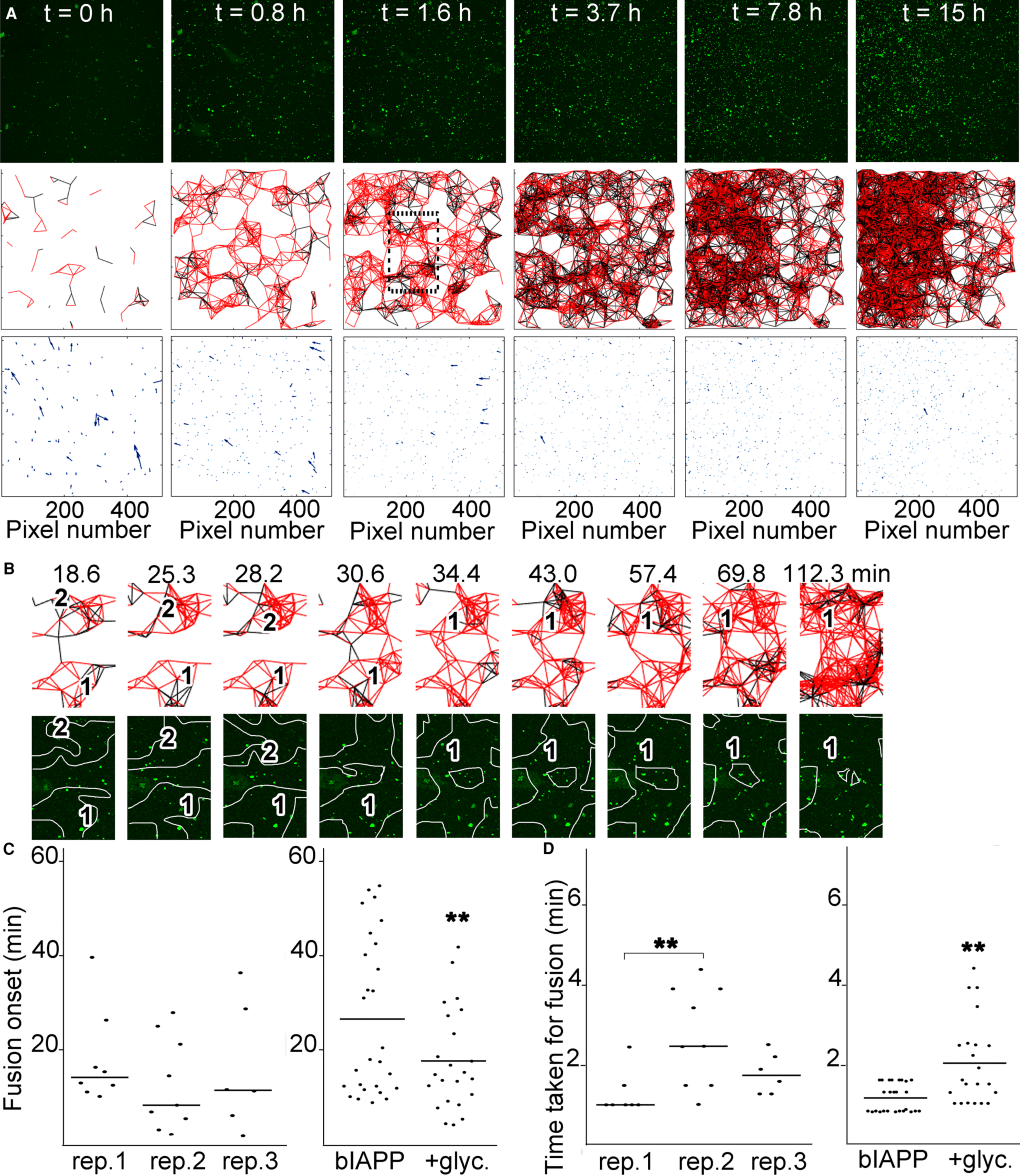
At the AWI, glycerol promotes the onset of meshwork formation and cluster fusion, but delays fusion completion. 3.6 μM IAPP–0.04 μM bIAPP–0.08 μM avidin D fluorescein in PBS was incubated with 6% glycerol in a well of a 96-well plate. The well was imaged, *z* stacks encompassing the AWI collected for at least 15 h, maximum intensity projections of the time course and aggregate tracking performed. From the aggregate tracking, movement vectors and connectivity between aggregates were determined. In (**A**) are shown maximum intensity projections of relevant time points of an 15 h time course (top panels), the connectivity between aggregates (a red line depicts aggregates moving in the same direction, i.e. connectedness; and a black line depicts aggregates moving in the opposite direction, i.e. non-connectedness)(middle panels), and the movement vectors (bottom panels). The dashed box is an area that is further examined in (**B**). (**B**) shows an example of aggregate connectivity over relevant time points (top panels), and the corresponding snapshots from a maximum intensity projection (bottom panels). The numbers within the projections indicate individual clusters of connected aggregates that evolve over time, and the white lines show the clusters of connected aggregates (the projection and connectedness were superimposed and lines were drawn around connected aggregates). (**C**) Quantitation of the timing taken for fusion event to occur for several fusion events of three independent replicates (rep.) in presence of glycerol (glyc.) (left panel). Comparison of the average timing of fusion onset for reactions with bIAPP alone, and bIAPP with glycerol (right panel). ** *P* < 0.03 when compared with bIAPP alone. (**D**) Quantitation of the time taken for the fusion to be complete for several fusion events of three independent replicates in presence of glycerol (right panel). *P *< 0.009. Comparison of the average time taken for fusion completion for reactions with bIAPP alone, and bIAPP with glycerol (right panel). ** *P* < 0.03 when compared with bIAPP alone.

We then assessed the effect of increased viscosity on the whole system. The presence of glycerol did not interfere with aggregate accumulation at the AWI, aggregate movement and connectedness, or cluster formation, as individual cluster of interconnected aggregates could be seen few minutes after the start of the reaction (Figure 6A). However, when looking at the evolution of the system over several hours, two differences were noticeable when compared with bIAPP alone. First, network formation occurred faster, ∼3.7–7.8 h to achieve a network as compact and as interconnected as that of bIAPP alone at ∼13.5–15 h. Second, at 15 h, the clusters had collapsed together into one large mass, rather than being dispersed clusters interconnecting each other as seen for bIAPP alone. Therefore, glycerol clearly affected how the clusters came together as meshwork arrangement/density occurred more rapidly, and the network was much compact than that of bIAPP.

When looking at the dynamics of cluster growth and fusion, individual aggregate cluster growth could clearly be observed, along with cluster growth (from 18.6 to 28.2 min), and cluster fusion (∼2.4 min, from 28.2 to 30.6 min) to form a larger cluster of interconnected aggregates, which carried on growing and evolving (Figure 6B). Systematic quantitation revealed that glycerol accelerated the onset of cluster fusion by 1.7 fold (15.72 vs 26.95 min, *P *< 0.009), and delayed fusion itself by 1.8 fold (1.94 vs 1.11 min, *P *< 0.012) (Figure 6C and Supplementary Figure S4). Moreover, glycerol exacerbated heterogeneity of cluster fusion, as in contrast with bIAPP alone, the variation between replicates was not as pronounced but there was more variation within replicates. For fusion onset, all three replicates showed pronounced variation within replicates, compared with mostly one replicate (replicate 2) for bIAPP alone. This suggests that glycerol introduced kinetic variation within replicates. For fusion completion, again all replicates showed significant variation within replicates, compared with none for bIAPP alone (Figure 6D and Supplementary Figure S4). This suggests that glycerol interfered with fusion itself.

### At the AWI, dextran promotes homogeneity, and delays cluster fusion

**Figure 7.**
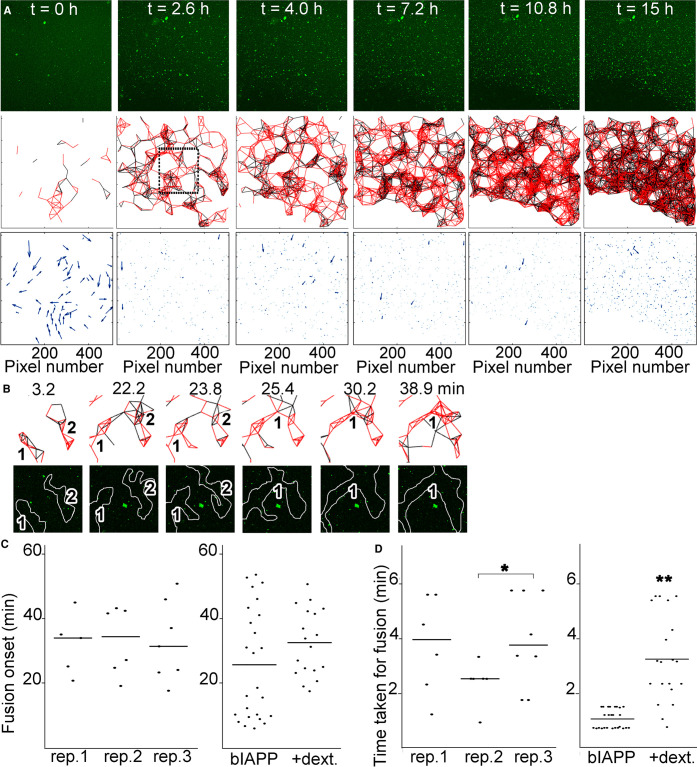
At the AWI, dextran delays fusion itself, but increases meshwork density. 3.6 μM IAPP–0.04 μM bIAPP–0.08 μM avidin D fluorescein in PBS was incubated with 6% dextran in a well of a 96-well plate. The well was imaged, *z* stacks encompassing the AWI collected for at least 15 h, maximum intensity projections of the time course and aggregate tracking performed. From the aggregate tracking, movement vectors and connectivity between aggregates were determined. In (**A**) are shown maximum intensity projections of relevant time points of an 15 h time course (top panels), the connectivity between aggregates (a red line depicts aggregates moving in the same direction, i.e. connectedness; and a black line depicts aggregates moving in the opposite direction, i.e. non-connectedness) (middle panels), and the movement vectors (bottom panels). The dashed box is an area that is further examined in (**B**). (**B**) shows an example of aggregate connectivity over relevant time points (top panels), and the corresponding snapshots from a maximum intensity projection (bottom panels). The numbers within the projections indicate individual clusters of connected aggregates that evolve over time, and the white lines show the clusters of connected aggregates (the projection and connectedness were superimposed and lines were drawn around connected aggregates). (**C**) Quantitation of the timing taken for fusion event to occur for several fusion events of three independent replicates (rep.) in presence of dextran (dext.) (left panel). Comparison of the average timing of fusion onset for reactions with bIAPP alone, and bIAPP with dextran (right panel). (**D**) Quantitation of the time taken for fusion to be complete for several fusion events of three independent replicates in presence of dextran (left panel). * *P *< 0.05. Comparison of the average time taken for fusion onset for reactions with bIAPP alone, and bIAPP with dextran (right panel). ** *P *< 0.03 when compared with bIAPP alone.

We then investigated the effect of macromolecular crowding, first by using dextran. Dextran did not interfere with aggregate accumulation at the AWI, aggregate movement and connectedness, or cluster formation, as individual aggregate clusters could be seen few minutes after the start of the reaction (Figure 7A). The timing of network formation was similar to that of bIAPP alone. However, dextran clearly affected how the clusters interacted as they form a meshwork more compact and interconnected than that of bIAPP alone, but less compact than that in presence of glycerol.

When examining the dynamics of cluster fusion, clear fusion could only be seen later than for bIAPP alone. In Figure 7B, two individual aggregate clusters were detected and fused together in ∼1.6 min (from 23.8 to 25.4 min) to generate one large cluster, which carried on growing. Systematic quantitation revealed that dextran delayed the onset of cluster fusion by 1.2 fold (32.79 vs 26.95 min, not statistically significant), and also delayed fusion itself by 2.6 fold (2.94 vs 1.11 min, *P *< 0.002) (Figure 7C,D, and Supplementary Figure S4). Moreover, and as seen for hydrogelation, dextran reduced the heterogeneity of the system, with only one kinetic regime identified. Indeed, the timings of fusion onset were similar between replicates, with an overall variation less than that of bIAPP alone (SEM of 9.38 vs 16.90 min). However, the variation within replicates was higher than that of bIAPP alone. The time taken for fusion to be complete was variable between (statistical significance between replicate 2 and 3, *P *< 0.05) and within replicates, suggesting that dextran interfered with the fusion process.

### At the AWI, Ficoll promotes homogeneity, and affects cluster fusion

**Figure 8.**
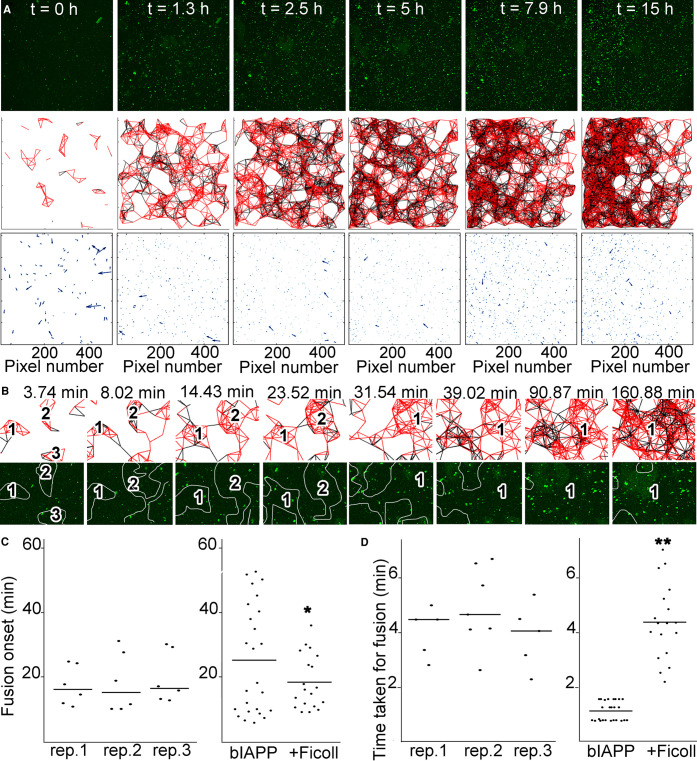
At the AWI, Ficoll promotes the onset of cluster fusion and delays fusion itself, but also increases the onset of meshwork formation and meshwork density. 3.6 μM IAPP–0.04 μM bIAPP–0.08 μM avidin D fluorescein in PBS was incubated with 6% Ficoll400 in a well of a 96-well plate. The well was imaged, *z* stacks encompassing the AWI collected for at least 15 h, maximum intensity projections of the time course and aggregate tracking performed. From the aggregate tracking, movement vectors and connectivity between aggregates were determined. In (**A**) are shown maximum intensity projections of relevant time points of an 15 h time course (top panels), the connectivity between aggregates (a red line depicts aggregates moving in the same direction, i.e. connectedness; and a black line depicts aggregates moving in the opposite direction, i.e. non-connectedness) (middle panels), and the movement vectors (bottom panels). The dashed boxes are areas that are further examined in (**B**). (**B**) shows an example of aggregate connectivity over relevant time points (top panels), and the corresponding snapshots from a maximum intensity projection (bottom panels). The numbers within the projections indicate individual clusters of connected aggregates that evolve over time, and the white lines show the clusters of connected aggregates (the projection and connectedness were superimposed and lines were drawn around connected aggregates). (**C**) Quantitation of the timing taken for fusion event to occur for several fusion events of three independent replicates (rep.) in presence of Ficoll (left panel). Comparison of the average timing of fusion onset for reactions with bIAPP alone, and bIAPP with Ficoll (right panel). * *P *< 0.05 when compared with bIAPP alone. (**D**) Quantitation of the time taken for the fusion to be complete for several fusion events of three independent replicates in presence of Ficoll (left panel). Comparison of the average time taken for fusion onset for reactions with bIAPP alone, and bIAPP with Ficoll (right panel). ** *P *< 0.03 when compared with bIAPP alone.

Finally, we assessed the effect of Ficoll. Ficoll did not interfere with aggregate accumulation at the AWI, aggregate movement and connectedness, or cluster formation, as individual clusters could be seen few minutes after the start of the reaction (Figure 8A). The clusters grew to form a macroscopic network, which started being evident ∼7.9 to 15 h, a timing similar to that with glycerol. In contrast with bIAPP alone, but similar to reactions containing glycerol or dextran, the aggregate clusters also collapsed into one large mass to form the meshwork.

When examining the dynamics of cluster fusion, clear fusion could only be seen earlier than for bIAPP alone. In Figure 8B, three individual aggregate clusters were observed. Two of these clusters (2 and 3) fused together in ∼4.3 min (from 3.74 to 8.02 min). The two remaining clusters (1 and ‘new 2’) carried on growing (from 8.02 to 23.52 min) before fusing together in ∼8 min (from 23.52 to 31.54 min) to generate one cluster that carried on evolving. Systematic quantitation revealed that Ficoll promoted the onset of cluster fusion by 1.4 fold (18.84 vs 26.95 min, *P *< 0.033), and delayed fusion itself by 3.9 fold (4.41 vs 1.11 min, *P *< 0.000002) (Figure 8C,D and Supplementary Figure S4). Moreover, and as seen for hydrogelation, Ficoll reduced the heterogeneity of the system, with only one kinetic regime identified. Indeed, the timings of fusion onset were similar between replicates, with an overall variation less than that of bIAPP alone (SEM of 8.59 vs 16.90 min). The variation within replicates was comparable or smaller than that of bIAPP alone, suggesting that Ficoll did not interfere with the overall kinetics leading to fusion onset. The time taken for fusion to be complete was also similar between but varied within replicates, suggesting that Ficoll shortened the time to fusion onset but slowed the fusion process itself, without affecting the eventual state of the system.

## Discussion

We previously demonstrated that hIAPP spontaneously undergoes AWI-catalysed LLPS and hydrogelation [29,32]. Hydrogelation of the phase-separated liquid droplets initiated amyloid aggregation and the formation of clusters of interconnected aggregates, which grew and fused to percolate the whole system. *In vitro*, crowding agents can change protein phase diagrams. However, the mechanisms underlying enhanced LLPS, and the effect(s) on stages beyond LLPS (i.e. hydrogelation via amyloid aggregation), remain poorly or not characterised. These stages are of critical importance as they are thought to be behind the pathology of several diseases [22,29–31]. Our paper focused on the role of macromolecular crowding in driving and shaping the stages beyond LLPS of hIAPP.

By rheology, we previously demonstrated that hIAPP hydrogelation followed two distinct kinetic regimes [32] Using live microscopy, we now show that the phase-separated aggregated IAPP system was also heterogeneous and governed by two distinct kinetic regimes. Indeed, the aggregate number, aggregate distribution, and the average aggregate speed varied between replicates; the onset of aggregate cluster fusion, fusion completion, and hydrogelation of the whole system, all revealed two distinct kinetic regimes. Although for fusion onset, variation was between and within replicates, fusion completion was only variable between but not within replicates. This suggests that once the aggregate clusters had converged, fusion occurred very quickly and homogeneously for all fusion events within a replicate. The varying number and distribution of aggregate clusters would affect the time taken for the clusters to encounter each other (leading to different timings for fusion onset), and eventually to percolate the whole system (leading to different timings for hydrogelation onset). For example, fewer aggregate clusters separated by longer distances would take longer to come together to fuse, leading to slower overall kinetics, and vice versa. From the timings of the first fusion events (26.9 ± 16.9 min on average) and of gelation onset (4.29 ± 1.05 h on average), we can infer that multiple cluster fusions were required before the whole system percolated. The kinetic variations of hIAPP hydrogelation in water were previously shown to be due to the system evolving independently from multiple pathways generated from varying number of phase-separated droplets/aggregate clusters, with eventually a few droplets/aggregate clusters coexisting with gelation occurring within them [29]. Our microscopy data corroborates this as individual aggregate clusters clearly evolve differently in term of aggregate speed, cluster fusion, and gelation.

We then addressed the effect of increased viscosity. Rheologically, increased viscosity introduced further kinetic variability of hydrogelation, did not affect gelation onset (although the trend indicated a delay), decreased gelation rate, and promoted the formation of a stronger hydrogel (increased viscoelasticity). Microscopically, increased viscosity also exacerbated the heterogeneity of hydrogelation (maximal across gelation onset compared with before gelation for IAPP alone), did not on average delay it, and promoted the more rapid formation of a more compact and interconnected meshwork than for IAPP alone, with the clusters collapsing together into one large mass rather than remaining more disperse. hIAPP LLPS, fibrillisation and hydrogelation are all dependent on adsorption to HHIs [10,29,32]. Increased viscosity would impede IAPP diffusion to the AWI, which would increase even more the kinetic variations of an already heterogeneous system, but also delay subsequent stages/steps reliant on diffusion such as aggregate cluster fusion and gelation. Although, the delay in gelation onset was not statistically significant, the onset was as slow as the ‘slow’ regime by rheology, and intermediate between the ‘slow’ and ‘fast’ by microscopy. However, the gelation rate was indeed decreased, and cluster fusion was heterogeneous with more variation between and within replicates than for IAPP alone. Increased viscosity also promoted the formation of a more rigid and stronger hIAPP hydrogel. A glycerol-induced increase in gelatine gel strength was previously observed [52]. The authors proposed that, by strongly H bonding with water, glycerol forced gelatine molecules into regions of glycerol-free H_2_O, which increased the local gelatine concentration, induced H bond formation between gelatine chains, and resulted in the formation of a stronger gel. Glycerol might have a similar effect on hIAPP gelation. Furthermore, glycerol can also stabilise amyloid fibrils [53]. A glycerol-induced reduction in fibril breakage might also contribute to the formation of a stronger IAPP gel network. The observed promotion of the onset of cluster fusion by increased viscosity would also be consistent with the formation of a stronger gelled meshwork. Although glycerol accelerated the onset of cluster fusion, it delayed fusion itself and exacerbated its heterogeneity. By promoting a stronger gel network, increased viscosity would promote aggregation from droplet surfaces that would interconnect more rapidly. This would lead to a more rapid onset of cluster fusion. Droplet collision depends on the viscosity of the system, and droplet coalescence dynamics can be slowed down by viscosity [54,55]. Although, we were investigating aggregate clusters formed from mature phase-separated droplets, rather than droplets, we propose that increased viscosity also affected completion of cluster fusion by decreasing the effective strength of intermolecular interactions between fibrillar aggregate clusters.

Both rheologically and microscopically, macromolecular crowding abolished the variation in gelation kinetics, with the system adopting an intermediate regime between ‘slow’ and ‘fast’. Both dextran70 and Ficoll400 were previously shown to promote LLPS via the excluded volume effect, which changed the protein phase boundary by sterically repulsing droplet-forming proteins and by being themselves excluded from the droplets [28,50,56]. We propose that the excluded volume effect, by reducing the available volume, increased the effective hIAPP concentration into small, localised and defined areas, which forces the system to be more constrained and less variable. Therefore, macromolecular crowding might eliminate the coexistence of multiple droplets/aggregate clusters and force the system to behave as a predominant one-droplet/aggregate cluster system.

Heterogeneous kinetics between individual, but identical, replicates and the existence of several aggregation pathways is not unique to IAPP, and has often been attributed to the stochastic nature of the nucleation step. For example, Hortschansky et al. [57] showed that identical samples of Aβ1–40 led to heterogeneous kinetics, mainly during nucleation. Similarly, polymerisation of sickle cell haemoglobin and β2-microglobulin exhibit stochastic variations during nucleation [58,59]. Molecular dynamics studies have also provided evidence that monomers can sample different conformations/pathways and stabilise aggregates of different structures, leading to heterogeneous kinetics of aggregation and multiple pathways [60]. In contrast, to dilute conditions, macromolecular crowding might favour or disfavour some of these different kinetic pathways, reducing the number of pathways available and resulting in less heterogeneity. For example, aggregates that are not tightly folded (i.e. needing more space) would be disfavoured. Munishkina et al. [61] investigated the fibrillisation kinetics of different types of aggregating proteins in the presence of various crowding agents. In the case of the Parkinson's disease-associated α-synuclein, which possesses several aggregating pathways, the authors found that macromolecular crowding considerably shifted the equilibrium of the system in favour of one of these pathways. Therefore, when aggregated species have a choice between several thermodynamically favourable pathways, macromolecular crowding can ‘force’ the system toward the single most thermodynamically favourable pathway, overall leading to a more homogeneous system, as we observed.

Ficoll did not promote hIAPP hydrogelation, promoted the onset of cluster fusion, and delayed fusion itself. Crowding-induced excluded volume effect can trigger droplet transition from liquid to gel by increasing the interconnection of the network within droplets. In turn, the exchange dynamics between bulk and droplets would be progressively abrogated, and droplet fusion almost arrested. A crowding (PEG) dependent progressive droplet transition from liquid to gel, and delay in droplet fusion, was observed for FUS and the nucleolar phosphoprotein NPM1 [62,63]. All hIAPP assembly stages are initially catalysed by the AWI, and rapid droplet hydrogelation and formation of an impermeable droplet coat promoted aggregation from the droplet surface and creation of a meshwork of aggregates interlinking droplets [29]. Ficoll is surface active and does not interact with hIAPP [38,42]. Therefore, Ficoll would adsorb into localised AWI patches by competing with IAPP. As the Ficoll meshwok expands at the AWI, it would compress the IAPP network leading to an increase in IAPP molecular packing. An increase of the number of IAPP molecules per unit area by compression of a Langmuir monolayer was previously demonstrated [64]. This increase in effective IAPP concentration in localised AWI areas would accelerate not only the already fast maturation of IAPP droplets, but also aggregation from the droplet surface, formation of aggregate clusters, and therefore the onset of cluster fusion. This is consistent with the formation of larger aggregate clusters in a shorter time frame than for IAPP alone, as observed by microscopy (Figures 5A and 8A). However, this faster aggregation could delay fusion completion as steric hindrance induced by fibrillar entanglement might impede the full merging of clusters.

Dextran did not promote IAPP hydrogelation and delayed cluster fusion. Dextran not being surface active, its crowding effect occurs in the bulk solution, which would be less effective as the AWI is the key catalyst for all IAPP assembly processes. Moreover, dextran is viscous and we demonstrated that increased viscosity delays hydrogelation. Therefore, any volume exclusion effect would be counteracted, to some extent, by the negative effect of viscosity, resulting in no promotion of hydrogelation. Furthermore, dextran was shown not to promote hydrogelation of the phase-separated droplets of a silk-spidroin like triblock fusion protein [65]. By not promoting hIAPP droplet hydrogelation, dextran would not affect the ensuing aggregation, aggregate cluster formation, and onset of cluster fusion, as observed. However, fusion itself could be slowed down by viscosity-induced diffusion impediment. Ficoll delayed fusion completion more than dextran (4.1 vs 2.7 fold difference to IAPP alone). Interstitial cavities within a crowder network could hinder fusion when the cluster size is larger than the cavities. Although dextran70 would occupy more volume than Ficoll400, it would behave as shorter polymer chains (as its molecular mass is ∼5.7 smaller than that of Ficoll400) creating larger cavities than Ficoll400, which would accommodate ‘larger’ clusters.

As mentioned above, dextran would exclude 1.1 times more volume than Ficoll. Indeed, 6% dextran70 would be 1.78 × 10^17^ molecules in the 345 μl reaction volume, and 6% Ficoll400 would be 3.12 × 10^16^ molecules. The Stokes radius of dextran is 58 Å and that of Ficoll is 100 Å. Therefore, the 1.78 × 10^17^ dextran molecules occupy 145 μl in a 345 μl reaction (1.78 × 10^17^ × (4*π*/3) × (5.8)^3^), whereas the 3.12 × 10^16^ Ficoll molecules occupy 131 μl. Therefore, the effective concentration of IAPP in a solution with 6% dextran70 would be 6.9 μM, and in a solution of 6% Ficoll400 would be 6.5 μM. Recently, we found that increasing the IAPP concentration accelerated the onset of LLPS [29]. The lowest IAPP concentrations used were 4 (as used in the current manuscript) and 8 μM, with the calculated effective IAPP concentrations, in presence of dextran or Ficoll, falling between. Furthermore, we have also previously investigated the effect of IAPP concentrations (from 4 to 8 μM) on its fibrillisation, and found that the dependence of IAPP nucleation on the monomer concentration is only evident in the absence of an AWI [66]. The lag phases for every IAPP concentrations tested in presence of an AWI, with multiple replicates per concentration, were identical and showed no heterogeneity. Since nucleation is the most stochastic steps in IAPP assembly, and noting that assays described here were done in the presence of an AWI, we would expect that increasing the effective IAPP concentration, by the addition of dextran or Ficoll, would lead to the system being more homogeneous, as observed. However, we must stress out that the effect of the crowders would not be as simple as just increasing the effective concentration of IAPP. Indeed, dextran would also increase viscosity, which would have a negative effect, and Ficoll would compete with IAPP for the AWI, which also would have a negative effect, as discussed above.

## Conclusions

In this paper, we demonstrate that kinetic heterogeneity of the stages beyond LLPS were exacerbated by increased viscosity but abolished by crowding. These results not only highlight the under-appreciated effect of macromolecular crowding in *in vitro* assays, but more importantly the effect that an *in vivo* crowded environment may have. Indeed, a crowded environment would influence the kinetics of LLPS, aggregation and hydrogelation. *In vivo*, adsorption to and hydrogelation on the surface of cellular membranes would drastically affect membrane integrity and generally cell functions. Furthermore, the presence of macromolecules, or changes in their expression levels, in the vicinity of polypeptides able to undergo LLPS could significantly affect their phase boundary, their concentration in the droplets, and droplet properties. *In vivo*, entropic forces of the crowded cellular environment would continuously tune the droplet physical properties (liquid, gel, impermeable coat) and influence the outcome of stages beyond LLPS. These changes would be critical for aggregation and pathology, as droplet hydrogelation promotes pathological amyloid aggregation and needs to be fine-tuned [28–30]. Moreover, changes in droplet properties could stop aggregation regulators entering the droplets, and this is important for understanding the mechanisms modulating stages beyond LLPS and, crucially, the limitations to therapeutic intervention in these processes.

## Data Availability

All the data presented in this paper are available to readers in the paper (main text or Supplementary Appendix).

## Open Access

Open access for this article was enabled by the participation of University of Oxford in an all-inclusive *Read & Publish* pilot with Portland Press and the Biochemical Society under a transformative agreement with JISC.

## Abbreviations

AWI: air–water interface
D2M: type II diabetes mellitus
FUS: fused in sarcoma
HHI: hydrophobic–hydrophilic interface
IAPP: islet amyloid polypeptide
LLPS: liquid–liquid phase separation
PBS: phosphate-buffered saline
PEG: polyethylene glycol
SD: standard deviation
SEM: standard error of the mean
