# Supplementary material for: The kinetics of islet amyloid polypeptide phase-separated system and hydrogel formation are critically influenced by macromolecular crowding
Source: Biochem J. 2021 Aug 13;478(15):3025–46. doi: 10.1042/BCJ20210384 (PMC8370757; doi:10.1042/BCJ20210384)
Supplement: Supplementary Figures S1-S4 [file BCJ-478-3025-s1.pdf]

SUPPLEMENTAL MATERIAL

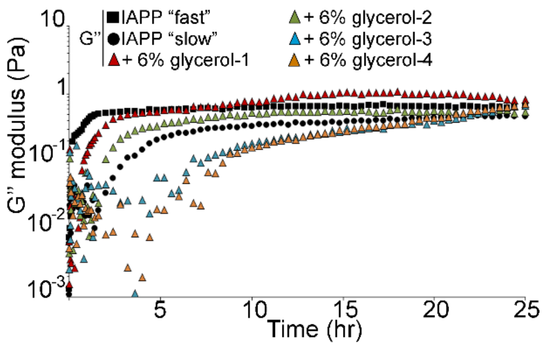

Fig. S1. Rheological properties of a 4 μM IAPP solution in presence of 6% glycerol were assessed at 25ºC with a controlled displacement of $5x10^{-3}$ rads and a frequency of 0.5 Hz. Dynamic modulus G'' as a function of time. The mean of at least three independent assays is shown for IAPP alone, and in presence of glycerol all the 4 independent replicates are shown.

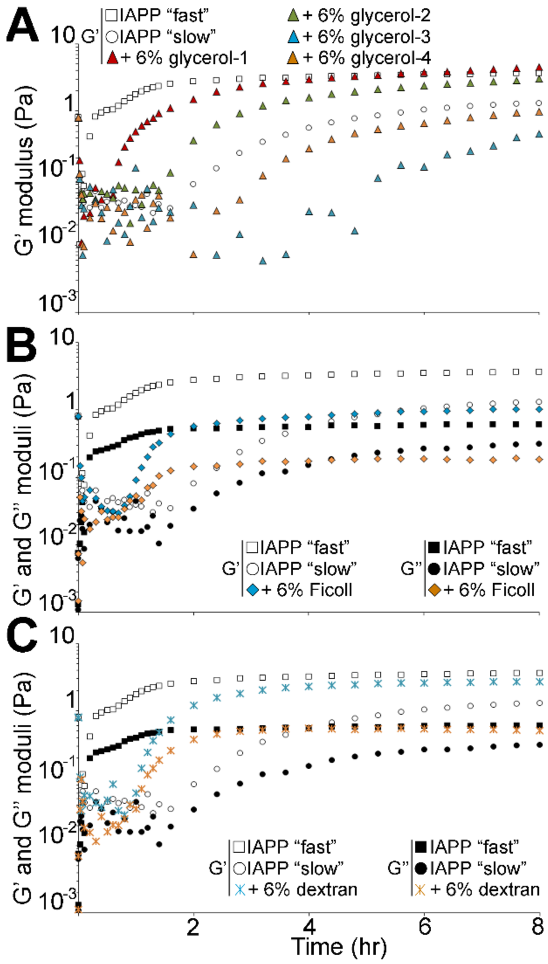

Fig. S2. Lag times of the gelation processes. Zoom in on the lag phases of the 3D rheology of a 4 µM IAPP solution in presence of 6% glycerol (**A**), 6% dextran (**B**) and 6% Ficoll (**C**). Dynamic moduli G' and G'' as a function of time are represented, except for glycerol for which only G' is presented for clarity. The mean of at least three independent assays is shown, except in presence of glycerol for which all the 4 individual and independent replicates are shown.

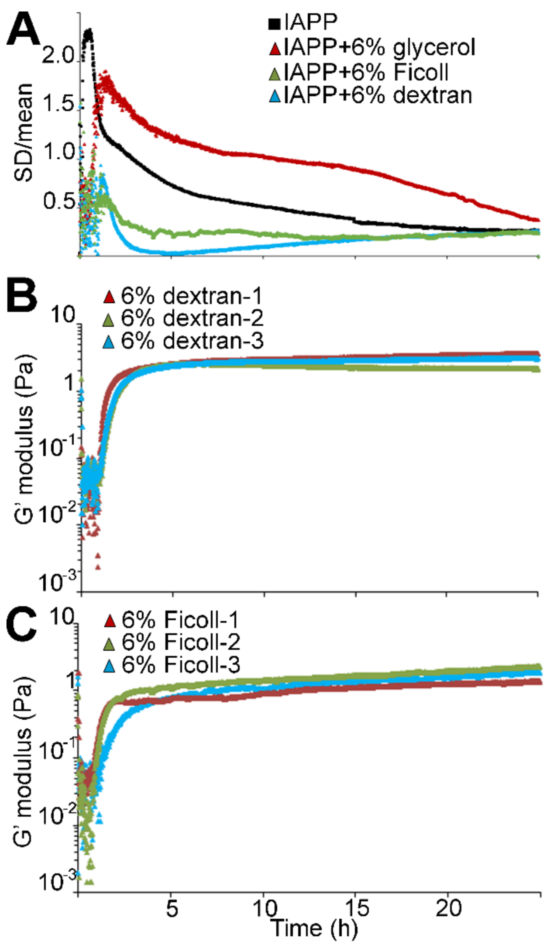

Fig. S3. Rheological properties of a 4 μM IAPP solution alone or in presence of 6% glycerol, 6% dextran, or 6% Ficoll were assessed at 25ºC with a controlled displacement of $5x10^{-3}$ rads and a frequency of 0.5 Hz. (**A**) Variation coefficient (SD over mean for G') over time. (**B**) and (**C**) Viscoelastic modulus G' as a function of time. Three independent assays are shown for IAPP in presence of dextran (**B**) or Ficoll (**C**).

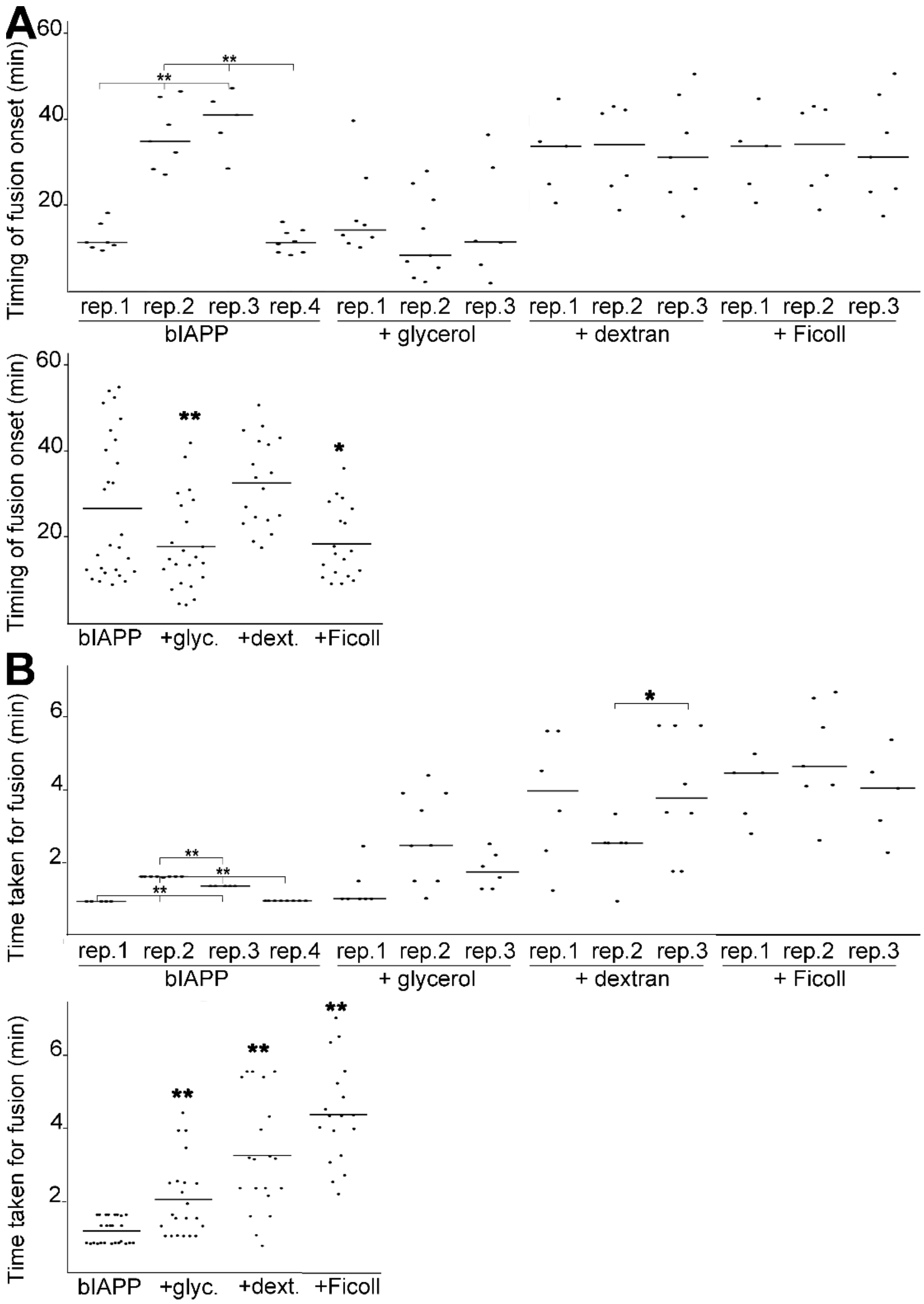

Fig. S4. At the AWI, Ficoll promotes the onset of cluster fusion and delays fusion itself, but also increases the onset of meshwork formation and meshwork density
Quantitation of the timing taken for fusion event to occur for several fusion events of independent replicates (rep.) for bIAPP alone, or for bIAPP in the presence of glycerol (glyc.), dextran (dext.), or Ficoll (top panel). ** $p<0.0006$. Comparison of the average timing of fusion onset for reactions with bIAPP alone, or bIAPP with glycerol, dextran, or Ficoll (bottom panel). ** $p<0.03$, * $p<0.05$. (**B**) Quantitation of the time taken for fusion to be complete for several fusion events of independent replicates for bIAPP alone, or for bIAPP in the presence of glycerol (glyc.), dextran (dext.), or Ficoll (top panel). ** $p<0.0006$, * $p<0.05$. Comparison of the average time taken for fusion onset for reactions with bIAPP alone, or bIAPP with glycerol, dextran, or Ficoll (bottom panel). ** $p<0.03$ when compared to bIAPP alone.

Movies

Movie 1. hIAPP phase separated system at the AWI - glycerol. 3.6 μM IAPP-0.4 μM bIAPP-0.08 μM avidin D fluorescein was incubated with 6% glycerol in H_2O in a well of a 96-well plate. The well was imaged, z stacks encompassing the AWI and an area spanning 1417x1417 μm^2 were collected for at least 15 h. 3D projections were performed in Imaris and visualised with the AWI at the front facing the experimentalist and the bulk solution behind it.

Movie 2. hIAPP phase separated system at the AWI - dextran. 3.6 μM IAPP-0.4 μM bIAPP-0.08 μM avidin D fluorescein was incubated with 6% dextran in H_2O in a well of a 96-well plate. The well was imaged, z stacks encompassing the AWI and an area spanning 1417x1417 μm^2 were collected for at least 15 h. 3D projections were performed in Imaris and visualised with the AWI at the front facing the experimentalist and the bulk solution behind it.

Movie 3. hIAPP phase separated system at the AWI - Ficoll. 3.6 μM IAPP-0.4 μM bIAPP-0.08 μM avidin D fluorescein was incubated with 6% Ficoll in H_2O in a well of a 96-well plate. The well was imaged, z stacks encompassing the AWI and an area spanning 1417x1417 μm^2 were collected for at least 15 h. 3D projections were performed in Imaris and visualised with the AWI at the front facing the experimentalist and the bulk solution behind it.

Movie 4. hIAPP phase separated system at the AWI - dextran. 3.6 μM IAPP-0.4 μM bIAPP-0.08 μM avidin D fluorescein was incubated with 6% dextran in H_2O in a well of a 96-well plate. The well was imaged, z stacks encompassing the AWI and an area spanning 1417x1417 μm^2 were collected for at least 15 h. 3D projections were performed in Imaris and visualised with the AWI at the front facing the
